# miR-185 Plays an Anti-Hypertrophic Role in the Heart via Multiple Targets in the Calcium-Signaling Pathways

**DOI:** 10.1371/journal.pone.0122509

**Published:** 2015-03-13

**Authors:** Jin Ock Kim, Dong Woo Song, Eun Jeong Kwon, Seong-Eui Hong, Hong Ki Song, Choon Kee Min, Do Han Kim

**Affiliations:** School of Life Sciences and Systems Biology Research Center, Gwangju Institute of Science and Technology (GIST), Gwangju, Korea; Faculty of Biochemistry, Biophysics and Biotechnology, Jagiellonian University, POLAND

## Abstract

MicroRNA (miRNA) is an endogenous non-coding RNA species that either inhibits RNA translation or promotes degradation of target mRNAs. miRNAs often regulate cellular signaling by targeting multiple genes within the pathways. In the present study, using Gene Set Analysis, a useful bioinformatics tool to identify miRNAs with multiple target genes in the same pathways, we identified *miR-185* as a key candidate regulator of cardiac hypertrophy. Using a mouse model, we found that *miR-185* was significantly down-regulated in myocardial cells during cardiac hypertrophy induced by transverse aortic constriction. To confirm that *miR-185* is an anti-hypertrophic miRNA, genetic manipulation studies such as overexpression and knock-down of *miR-185* in neonatal rat ventricular myocytes were conducted. The results showed that up-regulation of *miR-185* led to anti-hypertrophic effects, while down-regulation led to pro-hypertrophic effects, suggesting that *miR-185* has an anti-hypertrophic role in the heart. Our study further identified *Camk2d*, *Ncx1*, and *Nfatc3* as direct targets of *miR-185*. The activity of Nuclear Factor of Activated T-cell (NFAT) and calcium/calmodulin-dependent protein kinase II delta (CaMKIIδ) was negatively regulated by *miR-185* as assessed by NFAT-luciferase activity and western blotting. The expression of phospho-phospholamban (Thr-17), a marker of CaMKIIδ activity, was also significantly reduced by *miR-185*. In conclusion, *miR-185* effectively blocked cardiac hypertrophy signaling through multiple targets, rendering it a potential drug target for diseases such as heart failure.

## Introduction

Cardiac hypertrophy is an adaptive response to diverse extrinsic and intrinsic stimuli, which is accompanied by enlarged cardiomyocyte size, highly organized sarcomeres, increased protein synthesis and re-activation of fetal genes [1]. The hypertrophic gene program is complex and multifactorial, and it is continuously influenced by regulatory actions of genetic molecules such as miRNAs and mRNAs [2–4]. Although cardiac hypertrophy is initially compensatory to diverse stimuli, prolonged cardiac hypertrophy eventually leads to congestive heart failure and sudden death [5,6].

MicroRNA (miRNA) is a recently discovered species of non-coding RNAs that regulates target gene expression post-transcriptionally [7]. A number of recent experimental data have suggested that individual miRNAs modulate the expression of a set of genes that often share common signal transduction pathways in heart [8]. For example, *miR-378* targets the 3′-UTR of *Igf1r*, *Grb2*, *Ksr1*, and *Mapk1* to regulate the mitogen-activated protein kinase (MAPK) pathway [9]. *miR-133* protects the heart from apoptosis through direct repression of multiple key components along β1-adrenergic receptor signal transduction, such as *adbr1*, *adcy6*, *prkacb*, and *epac* [10]. Thus far, numerous miRNAs showing differential expression during pathological cardiac hypertrophy have been described [11,12], but whether those documented miRNAs are able to interact with multiple targets in the hypertrophic processes still not been systematically studied.

Gene Set Analysis (GSA) is a useful bioinformatics tool developed to identify signaling pathways for certain biological phenomena, by performing statistical enrichment tests for pre-defined gene sets. It is especially useful to infer functions of miRNAs, since numerous miRNA targets can be grouped into signaling pathways, allowing for interpretation of their functions.

In the present study, we performed cardiac specific GSA to identify specific miRNAs whose targets are significantly enriched in the hypertrophic signaling pathways, thereby efficiently regulating the gene programs. Among 18 candidate miRNAs, we found that *miR-185* plays a significant anti-hypertrophic role in the heart through multiple targets in Ca^2+^-signaling. We propose that *miR-185* is a potential drug target for diseases such as heart failure.

## Materials And Methods

### Aortic banding

The transverse aortic constriction (TAC) operation was performed on 8-week-old male C57BL/6 mice under anesthesia by intraperitoneal injection of avertin, 2–2–2 tribromoethanol (Sigma-Aldrich) dissolved in tert-amyl alcohol (Sigma-Aldrich). The operation procedure was followed as previously described [13]. The mice were anesthetized, and then were ventilated with a tidal volume of 0.1 mL and a respiratory rate of 120 breaths per min (Harvard Apparatus). The chest was opened and the transverse aortic arch was tied using a 27-gauge needle to give same space to aorta. Sham-operated mice were treated identically except the aorta was not ligated. On days 1, 3, 7, 14, 21, and 35 after the operation, mice were sacrificed by cervical dislocation, and hearts were removed, and weighted promptly.

### Cell culture and transfection

Neonatal rat ventricular myocytes (NRVMs) and neonatal rat cardiac fibroblasts were isolated using neonatal cardiomyocyte isolation system (Worthington), according to the manufacturer’s instructions. Ten or more hearts were removed from 1- to 3-day-old Sprague-Dawley rat pups, finely minced, and digested with trypsin overnight at 4°C. The following day, the heart tissue was dissociated by collagenase treatment (300 units/ml) at 37°C for 45 min. Cardiomyocytes and cardiac fibroblasts were separated using selective attachment procedures and were cultured in Dulbecco's modified Eagle's medium (DMEM) supplemented with 10% FBS (GIBCO). Cardiac fibroblasts were grown to confluence and subsequently passaged by trypsin. Quantitative real-time PCR (qRT-PCR) analysis of *miR-185* expression was conducted on cardiac fibroblasts at *passage* 3. Cardiomyocytes were seeded at a density of 0.9 million cells per dish onto 1% gelatin-coated 60-mm (Corning) culture dishes and cultured overnight in DMEM supplemented with 10% FBS, 1% antibiotics (WelGENE), 0.1 mmol/L BrdU at 37°C in a humidified incubator with 5% CO_2_. The following day, they were placed on serum-free medium without antibiotics for 24 h prior to miRNA transfection. Cells were then transfected with 15 nmol/L miRIDIAN microRNA mimic rno-miR-185, miRIDIAN microRNA mimic negative control #1 (NC), 100 nmol/L of miRIDIAN microRNA hairpin inhibitor rno-miR-185, and miRIDIAN microRNA hairpin inhibitor negative control #2 (NC inhibitor) using DharmaFECT-3 reagent (all obtained from Dharmacon) according to the manufacturer’s instructions. After 24 h, NRVMs were stimulated with hypertrophic reagent, endothelin-1 (ET-1, 10 nmol/L, Sigma-Aldrich) for 48 h, and cells were harvested for RNA isolation, immunocytochemistry, or western blot analysis. ET-1 was solubilized in oxygen free water to minimize oxidation.

### Total RNA preparation and reverse transcription

Total RNA and mature miRNAs were isolated from whole hearts, NRVMs, or cardiac fibroblasts using a miRNeasy Mini kit (Qiagen), and reverse transcribed with a miScript Reverse Transcription Kit (Qiagen) in accordance with the manufacturer’s instructions.

### qRT-PCR

qRT-PCR for *miR-185* targets and hypertrophic markers was performed with primers listed in Table B in S1 File, using SYBR green dye (Kapa Biosystem) and StepOne Plus Real Time PCR System (Applied Biosystems). miRNA-specific qRT-PCR in tissue or isolated cells was done using miScript SYBR Green PCR Kit (Qiagen) according to the manufacturer’s protocol, with miScript Primer Assay (for *miR-185*; Qiagen). The expression of the mRNAs was normalized to 18S rRNA and the level of *miR-185* was normalized to *U6* small RNA using the Hs_RNU6B_2 miScript Primer Assay (Qiagen). All reactions were performed in triplicate.

### Immunostaining and cell surface area measurements

NRVMs were grown on 1% gelatin coated-glass coverslips (18-mm diameter). The cells then were transfected with miRNA mimic or hairpin inhibitor for *miR-185*, or for the negative control, with DharmaFECT-3 reagent. Twenty four hours after transfection, cells were stimulated with 10 nmol/L ET-1 for another 48 h to induce cardiac hypertrophy. Next, cells were fixed with 4% paraformaldehyde for 15 min at RT, washed 3 times with PBS, permeabilized with 0.1% Triton X-100, and blocked with 10% bovine serum albumin (Sigma-Aldrich) in PBS for 30 min. The cells then were incubated with anti-α-actinin antibody (Sigma-Aldrich) in blocking buffer O/N at 4°C, rinsed six times with PBS, incubated with secondary antibody (Alexa 594-conjugated anti-mouse IgG antibody, Molecular Probe) in the blocking buffer for 2 h at 37°C, rinsed three times with PBS, and mounted. The slides were examined with an LSM 700 confocal laser scanning microscope (Carl Zeiss).

### Luciferase reporter assay

For miRNA target identification, we constructed reporter vectors bearing the exact target sites for *miR-185*. We obtained 250–350 base pairs of the 3′-UTRs of mouse target mRNAs by PCR amplification. The mutant constructs were generated by introducing mutations into the putative *miR-185*-binding sites by standard overlap PCR using mutagenic primers. All constructs were sequenced to confirm that the desired mutations had been obtained. To form a chimeric plasmid, the amplified products were inserted into the multiple cloning sites immediately downstream of the luciferase gene via the NheI and XhoI restriction sites in the pmirGLO Dual-Luciferase miRNA target expression vector (Promega). Then, human embryonic kidney (HEK)-293 cells were transfected using Lipofectamin LTX (Invitrogen) with 0.5 μg of the pmirGLO chimeric plasmid containing wild-type (WT) or mutant 3′-UTR along with the NC or *miR-185* mimic (Dharmacon) at a final concentration of 15 nmol/L. Seventy-two hours after transfection, cells were lysed and the reporter activity was detected with the Dual-Luciferase Reporter Assay System (Promega) on the Victor X3 multilabel plate reader (PerkinElmer, Waltham, MA). Firefly luciferase activity was normalized to the corresponding *Renilla* luciferase activity. For all experiments, transfection took place 24 h after starvation of cells in serum-free medium. The normalized luciferase activity relative to control group was used to demonstrate the alterations of mRNA transcription.

For measuring Nuclear Factor of Activated T-cell (NFAT) activity, NFAT-luciferase assays were performed as described previously with minor modifications [14]. Briefly, 9xNFAT-luciferase reporter plasmid and pRL-TK containing the *Renilla* luciferase gene were cotransfected into NRVMs 24 h after transfection of NC inhibitor or *miR-185* inhibitor. The next day, NRVMs were stimulated with ET-1 (10 nmol/L) for 24 h. A luciferase reporter plasmid driven by nine NFAT binding sites was kindly provided by Dr. Jeffery D. Molkentin (University of Cincinnati, Ohio).

### Western blotting

Protein samples were prepared from freshly isolated NRVMs using ice-cold lysis buffer supplemented with the protease inhibitor cocktail (Roche) and the phosphatase inhibitor cocktail (PhosSTOP, Roche). Protein concentrations were determined using the BCA protein assay kit (Pierce). Samples were subsequently separated by SDS-PAGE and transferred to polyvinylidene fluoride (PVDF) membranes followed by blocking with 5% skim milk (BD Science) or 5% BSA (Sigma-Aldrich) in TBST (0.1% Tween 20 in Tris-buffered saline; 137 mmol/L NaCl and 20 mmol/L Tris/HCl, pH 7.4) for 1 h at room temperature. Membranes were then incubated overnight at 4°C with the following antibodies: anti-CaMKIIδ (Santa Cruz), anti-phospho-CaMKIIδ (Cell Signaling), anti-NCX1 (Abcam), anti-NFATC3 (Santa Cruz), anti-p-NFATC3 (Santa Cruz), anti-phospholamban (Thermo Fisher Scientific), anti-phospho-phospholamban (Thr-17; Badrilla), anti-α-tubulin (Santa Cruz), and anti-GAPDH (homemade). After primary antibody incubation, membranes were washed with TBST and further incubated with the appropriate horseradish peroxidase-conjugated (HRP-conjugated) secondary antibody at room temperature for 1 h. The western blot signal was detected using an ImageQuant LAS 4000 mini (GE Healthcare Bio-Sciences AB) and a SuperSignal West Pico Chemiluminescence Kit (Thermo Fisher Scientific). The intensities of the protein bands were analyzed by ImageJ software (NIH).

### Ethical statement

All animal experiments were approved by the Gwangju Institute of Science and Technology Animal Care and Use Committee (Permit number: GIST2012–15).

### Statistical analysis

We used the Student’s unpaired *t*-test for comparisons between the two groups. Data are shown as mean ± SEM from more than 3 independent experiments conducted on separate days. *P*-values of *P<0.05 and **P<0.01 were considered statistically significant.

## Results

### Identification of candidate miRNAs associated with the cardiac hypertrophy signaling pathway

In the present study, we developed a novel framework to infer functions of cardiac miRNAs by employing 143 consensus cardiac-expressed miRNAs and 10,451 cardiac-expressed mRNAs (Figure A in S1 File and S1 Table). To strengthen our prediction power and infer more fundamental mechanisms of regulation by miRNA, we took into account common miRNA:mRNA complementarity in human and mouse, and context score percentile (CSP) of seed sites during the enrichment test. By doing so, we derived 902 miRNA-pathway connections in total (S2 Table). We explored the ability of our method to identify hypertrophy-modulating miRNAs by testing several hypertrophy-related gene sets extracted from databases. Strikingly, our results showed strong enrichment of targets of 18 miRNAs in various cardiac hypertrophy signaling pathways, including earlier investigations for the regulation of cardiac hypertrophy by miRNAs (Figure B in S1 File). For example, it has been reported that *miR-221* [15], *miR-199a/b* [16][17], *miR-27b* [18], *miR-195* [11] and *miR-34a/b/c* [19] positively regulate cardiac hypertrophy, while *miR-378* [9], *miR-29* [20], *miR-150* [11], *miR-223* [21] and *miR-1* [22] negatively regulate cardiac hypertrophy. On the other hand, *miR-99a* [23] and *miR-486* [24,25] are involved in myocardial remodeling through the regulation of different types of signaling pathways. Moreover, we found that each predicted miRNA family has multiple high-score targets in the cardiac hypertrophy signaling pathway (S2 Table), of which many already have been validated as direct targets of the corresponding miRNAs *in vitro*. Figure B in S1 File is a comparison of miRNA target enrichment results, showing a large overlap among the predicted miRNA-hypertrophy pathway associations. The integrated cardiac hypertrophy network (Figure C in S1 File) suggests potential interplay among the 18 miRNAs in the regulation of cardiac hypertrophy signaling pathway through highly intricate co-targeting.

### *miR-185* negatively regulates cardiac hypertrophy

**Fig 1:**
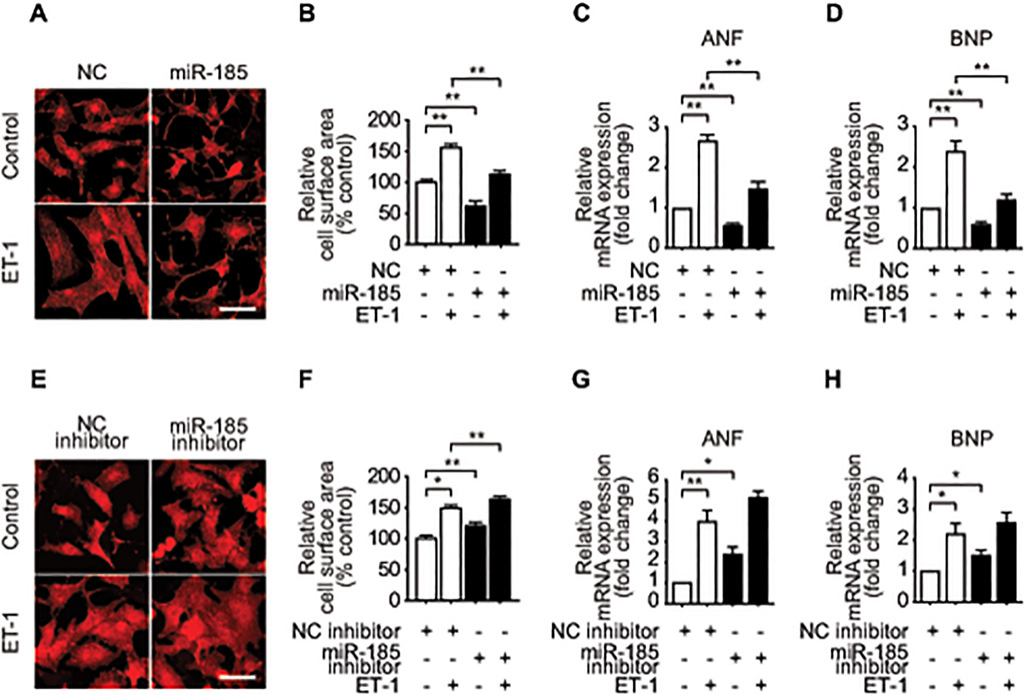
*miR-185* negatively regulate cardiomyocyte hypertrophy. (A, E) Microscopic images of immunofluorescence staining of NRVMs with α-actinin antibody. Twenty-four hours after transfection with *miR-185* (15 nmol/L) (A) or *miR-185* inhibitor (100 nmol/L) (E), NRVMs were stimulated with ET-1 for 48 h. Scale bar = 50 μm. (B, F) Cell surface areas of NRVMs measured using Image J software (N = 50 cells for each group). Untreated NRVMs served as controls. (C, D, G, H) qRT-PCR analysis of hypertrophic marker genes after transfection of *miR-185* mimic (C, D) or *miR-185* inhibitor (G, H) in the presence or absence of ET-1 (10 nmol/L). All data are expressed as mean ± SEM; *P < 0.05, **P < 0.001; N = 4.

Among the identified 18 miRNAs through GSA, six were previously unknown. We attempted to verify the effects of these six miRNAs (*miR-185*, *miR-139–5p*, *miR-374*, *miR-324–5p*, *miR-153*, and *miR-141*) on myocardial hypertrophy. qRT-PCR analysis of the hypertrophy markers ANF and BNP in transfected NRVMs showed that *miR-139–5p* and *miR-374* mimics markedly increased the expression of ANF and BNP, while *miR-324–5p*, *miR-153*, and *miR-141* mimics did not significantly affect the expression (unpublished data). However, transfection of *miR-185* mimic significantly reduced the mRNA expression of ANF and BNP in NRVMs (Fig. 1C and 1D).

*miR-185* was selected for further study due to the following reasons: First, *miR-185* was consistently found across four different cardiac hypertrophy signaling pathways. Second, *miR-185* is expressed primarily in the heart, brain and kidney [26], suggesting a potential role in these tissues. Third, *miR-185* is differentially expressed in the heart of pressure overload-induced cardiac hypertrophy models [12]. Finally, *miR-185* has 22 high score targets in cardiac hypertrophy signaling pathway, as determined by cross-species target predictions (Figure B in S1 File).

We first established a hypertrophy animal model by performing a severe TAC for 1, 3, 7, 14, 21, and 35 days. In this model, cardiac hypertrophy gradually developed and reached a peak on day 21 after TAC (Figure D in S1 File). To determine the level of *miR-185* during the progression of cardiac hypertrophy, we carried out qRT-PCR analysis. Consistent with the previous report [12], we found that the expression of *miR-185* was dramatically down-regulated after TAC, reached a minimal level on day 7 (Figure D in S1 File). The result suggests that *miR-185* may be a critical, quick-responder, regulating cardiac hypertrophy in response to increased biomechanical stress. We also found that the level of *miR-185* is approximately 2 fold higher in cardiomyocytes than cardiac fibroblasts, emphasizing the possible involvement of *miR-185* in cardiomyocyte hypertrophy (Figure D in S1 File).

To investigate the role of *miR-185* in cardiac hypertrophy, we transfected NRVMs with miRNA mimic or inhibitor of *miR-185*, followed by ET-1 stimulation. We found that transfection of *miR-185* reduced ET-1-induced cardiomyocytes hypertrophy, as assessed by the cell surface area measurement (Fig. 1A and 1B) and hypertrophic markers, including *ANF* and *BNP* (Fig. 1C and 1D). To further substantiate the inhibitory role of *miR-185*, we performed knockdown experiments. Treatment with a specific inhibitor for *miR-185* markedly down-regulated *miR-185* expression (Figure E in S1 File) and accelerated ET-1 induced cardiomyocytes hypertrophy, as shown in Figs. 1E-1H. The expression of *miR-185*, however, is not regulated by ET-1 itself (Figure F in S1 File). Similar anti-hypertrophic effects were observed when NRVMs were stimulated with other hypertrophic agents, such as isoproterenol (ISO, 10 μM) and phenylephrine (PE, 100 μM), after transfection of *miR-185*. As shown in Figure G in S1 File, over-expression of *miR-185* significantly inhibited ISO- or PE-induced cardiomyocyte hypertrophy as assessed by cell surface area and hypertrophic marker gene expressions. Collectively, the results indicate that *miR-185* negatively regulates cardiac hypertrophy.

### *miR-185* directly targets multiple key components in the calcium-activated hypertrophic signaling pathway

**Fig 2:**
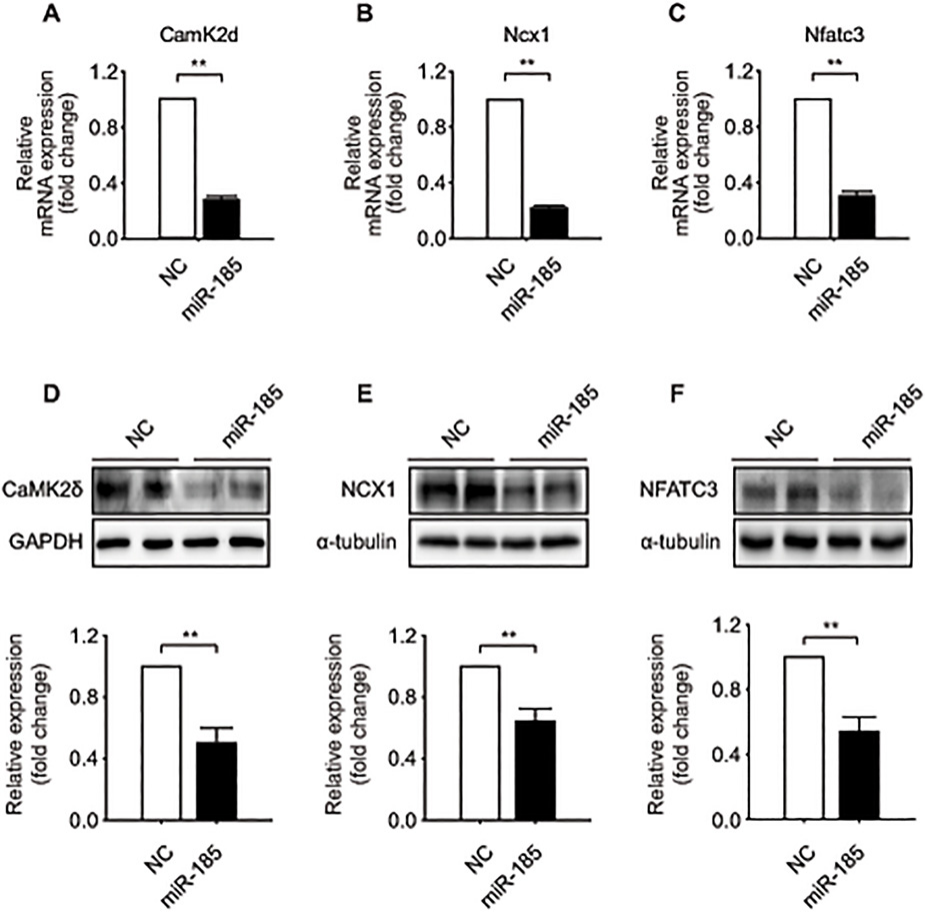
*miR-185* regulates the expression of CaMKIIδ, NCX1, and NFATC3 in cultured NRVMs. (A-C) qRT-PCR analyses of *Camk2d*, *Ncx1* and *Nfatc3* expression after transfection with *miR-185* or NC. (D-F) *miR-185* transfected NRVMs were lysed and analyzed by western blotting using antibodies against the proteins of interest. GAPDH or α-tubulin was used as a loading control. Representative western blots (upper) and quantified western blots (bottom). Data represent the mean ± SEM; *P < 0.05, **P < 0.001, N = 4.

We next screened for components in the cardiac hypertrophy signaling cascade that are controlled by *miR-185*. Several high score predicted targets were chosen for validation as key mediators of cardiac hypertrophy based on GSA (Figure B in S1 File). To confirm these target predictions, we transfected *miR-185* into NRVMs and determined whether endogenous levels of those target genes were down-regulated. The results showed that both mRNA and protein expression levels of calcium/calmodulin-dependent protein kinase II delta (CaMKIIδ), Na^+^-Ca^2+^ exchanger gene (NCX1/SLC8A1), and NFATC3 were significantly repressed by *miR-185* compared with expression in the controls (Fig. 2A-F).

**Fig 3:**
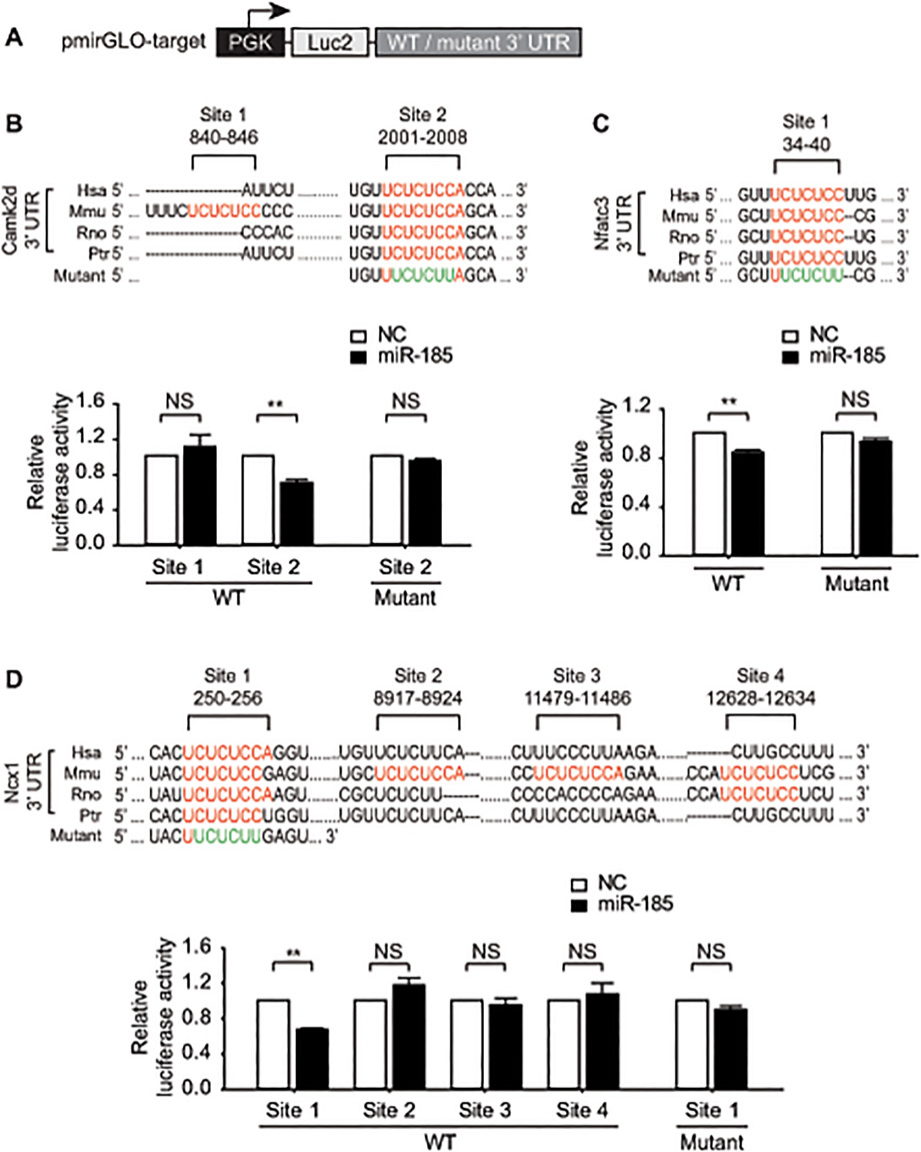
*miR-185* directly targets 3′-UTR of *Camk2d*, *Ncx1*, and *Nfatc3*. (A) Schematic diagram of the pmirGLO chimeric vector indicating where the exact complement target sequences for *miR-185* or mutant sequences were cloned into the 3′-UTR of the luciferase gene. (B-D) The relative positions of predicted binding sites for mouse *miR-185* in the 3′-UTR of the target mRNAs (upper) and quantitative analysis of luciferase activity of the reporter constructs (bottom). HEK-293 cells were transfected with *miR-185* in addition to the reporter constructs with WT or mutated 3′-UTR. After 72 h following transfection, cells were lysed for dual luciferase assay. Hsa, human; Mmu, mouse; Rno, rat; Ptr, chimpanzee. Data represent the mean ± SEM; **P < 0.001, or NS (not significant), N = 3.

Next, we determined whether *Camk2d*, *Ncx1*, and *Nfatc3* are the direct targets of *miR-185*. While *Nfatc3* harbors a single binding site for *miR-185* in the 3′-UTR (Fig. 3C), there are multiple putative binding sites in the 3′-UTRs of *Camk2d* and *Ncx1* in mouse (Fig. 3B and 3D). To critically examine the interactions between *miR-185* and the putative targets, we measured the activity of the luciferase reporter when linked to either the WT or mutant 3′-UTR of each target (Fig. 3A). The results showed that luciferase activity at Site 2 of *Camk2d*, and at Site 1 of *Nfatc3* and *Ncx1*, which are highly conserved across species, was significantly suppressed by *miR-185*. In the same context, mutations of the target sites in the 3′-UTR completely blocked the inhibitory effects of *miR-185*, suggesting that the identified sites, shown in Figs. 3B-3D, are the direct target sites for *miR-185* regulation. Other predicted targets, including *Ctf1*, *Elk1*, and *Mtpn*, were false positives (Figure H in S1 File). Collectively, our results suggest that *miR-185* have multiple targets in the Ca^2+^-dependent cardiac hypertrophy signaling pathway.

### *miR-185* regulates NFAT activity *in vitro*

**Fig 4:**
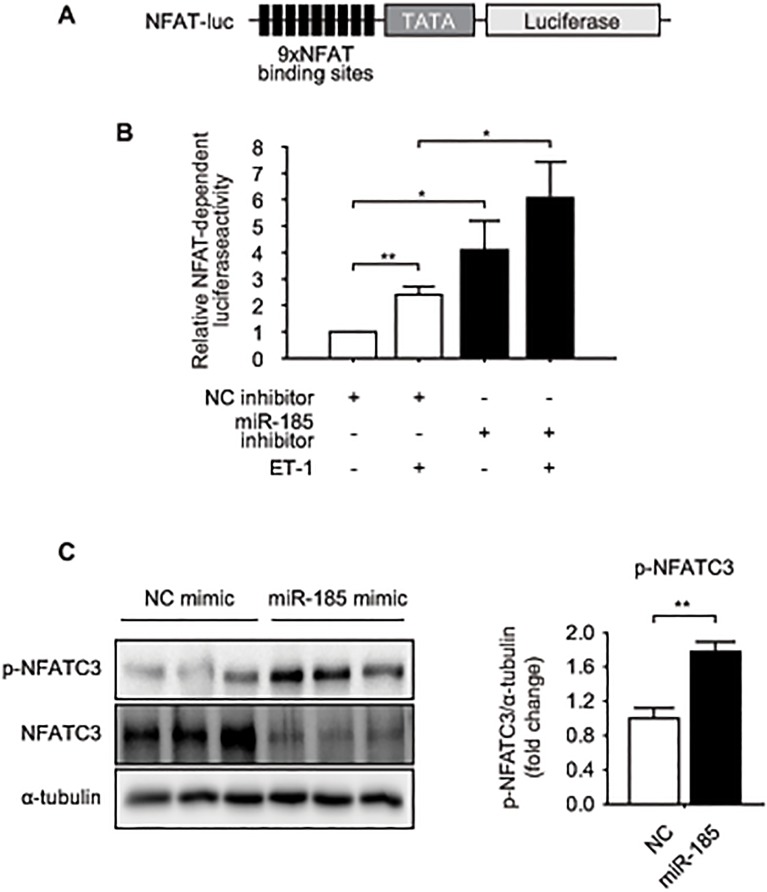
*miR-185* negatively regulates NFAT activity in NRVMs. (A) Schematic representation of the luciferase reporter construct driven by nine tandem NFAT binding sites. (B) Relative luciferase activity in NRVMs transfected with NC or *miR-185* inhibitor. NRVMs were stimulated by ET-1 (10 nmol/L) for 24 h. pRL-TK was transfected for normalization and as an internal control for transfection efficiency. (C) 72 h after transfection of NC or *miR-185* mimic, the level of p-NFATC3 and total NFATC3 were analyzed by western blotting. α-tubulin was used as a loading control. Representative western blots (left) and quantified western blots (right). The data are expressed as mean ± SEM of more than three independent experiments; *P < 0.05, **P < 0.001.

Based on evidence concerning the regulation of calcineurin-NFAT signaling by *miR-185* (Fig. 3C, Figures B and J in S1 File), we further examined whether inhibition of *miR-185* could activate calcineurin-dependent NFAT transcription factor by luciferase reporter assay employing a reporter under transcriptional control of nine NFAT binding sites (Fig. 4A). Transfection of NRVMs with *miR-185* inhibitor led to marked induction of the NFAT-dependent luciferase activity compared with the controls for the basal state and for the ET-1 stimulation (Fig. 4B). In contrast, overexpression of *miR-185* significantly induced phosphorylation of NFATC3, while significantly decreasing the total NFATC3, as assessed by western blotting (Fig. 4C), suggesting that *miR-185* negatively regulates calcineurin-NFAT signaling.

### *miR-185* modulates CaMKIIδ activity *in vitro*

**Fig 5:**
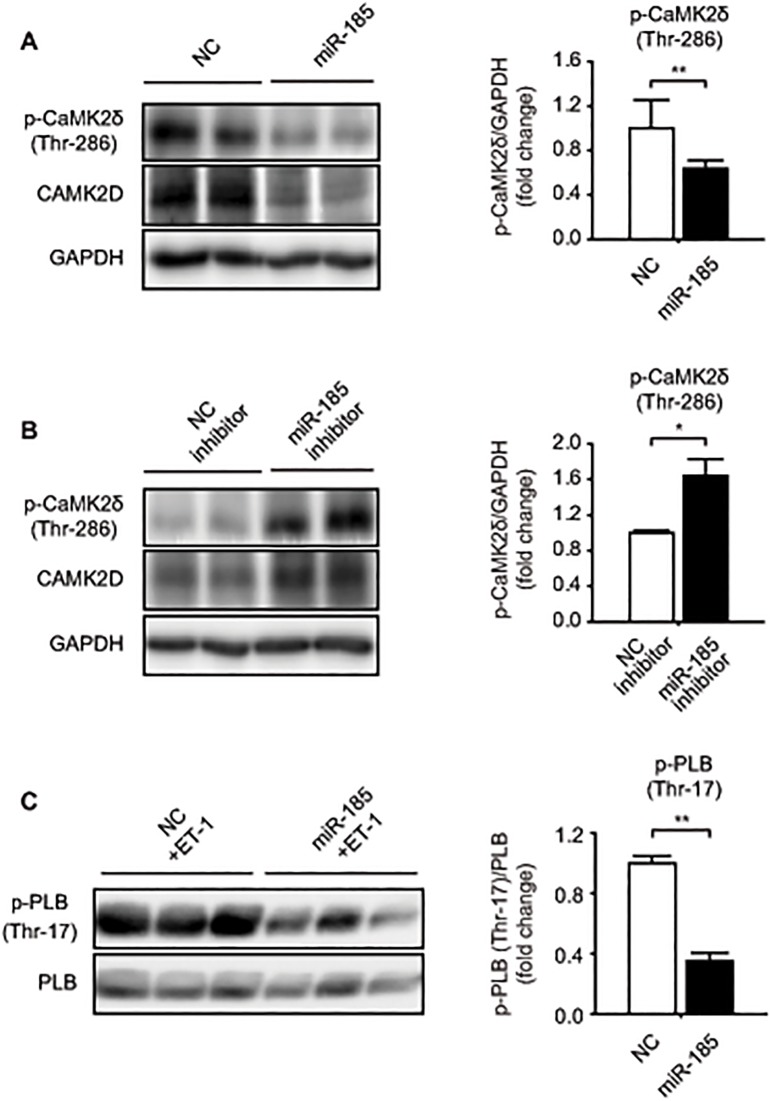
*miR-185* negatively regulates the activity of CaMKIIδ in NRVMs. (A and B) 72 h after transfection of *miR-185* mimic or *miR-185* inhibitor, the level of p-CaMKIIδ and total CaMKIIδ were analyzed by western blotting. GAPDH was used as a loading control. (C) Western blotting showing p-PLB (Thr-17) and PLB protein expression in NRVMs transfected with NC or *miR-185*. 24 h after transfection, NRVMs were stimulated with ET-1 (10 nmol/L) for 48 h. The blots were stripped for 30 min and reprobed with PLB for loading control. The data are expressed as mean ± SEM; *P < 0.05, **P < 0.001, N = 3.

Binding of Ca^2+^/calmodulin to the regulatory domain leads activates CaMKIIδ, and the activated enzyme is subsequently autophosphorylated at Thr-286/287, rendering the kinase constitutively active [27]. Based on our identification of *Camk2d* as a target of *miR-185* (Fig. 3B), we performed western blotting to examine the level of p-CaMKIIδ after transfection of *miR-185* mimic or *miR-185* inhibitor. As expected, the levels of CaMKIIδ phosphorylation at Thr-286 and total CaMKIIδ were significantly lower in *miR-185*-overexpressing cardiomyocytes compared with the levels in the control group (Fig. 5A), while inhibition of *miR-185* significantly up-regulated phosphorylation of CaMKIIδ at Thr-286 and the amount of total CaMKIIδ (Fig. 5B).

Since Thr-17 and Ser-16 of phospholamban (PLB) are independently phosphorylated by CaMKIIδ and cAMP-dependent protein kinase (PKA), respectively [28], we assessed phosphorylation of PLB at Thr-17 as an index of endogenous CaMKIIδ activity in NRVMs after transfection of *miR-185* mimic or NC mimic. Consistent with the change in CaMKIIδ autophosphorylation at Thr-286, overexpression of *miR-185* significantly reduced phosphorylation of PLB at Thr-17 compared with control group (Fig. 5C), demonstrating that *miR-185* is directly involved in the modulation of the CaMKIIδ activity.

## Discussion

**Fig 6:**
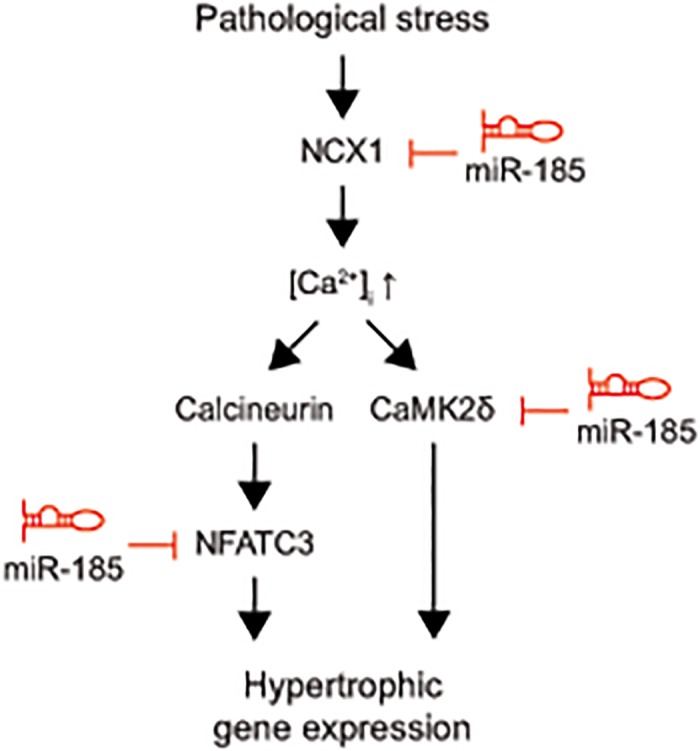
Working model illustrating the multiple targets of *miR-185* in the calcium-dependent cardiac hypertrophy signaling pathway.

Cardiac hypertrophy is a complex gene regulatory disorder that involves aberrant expression of coding mRNAs and regulatory non-coding RNAs. Among the noncoding RNAs, miRNA is the most widely studied one. It is now generally accepted that miRNAs often regulate multiple transcripts in the same biological processes [8]. However, the traditional approach has mostly focused on the identification of a single target for the specific miRNAs in different tissues. In the present study, we used cardiac tissue specific GSA to identify the critical miRNAs and their multiple targets involved in cardiac hypertrophy. Among the 18 GSA-identified cardiac hypertrophy-associated miRNAs, we selected *miR-185* for further studies. We found that *miR-185* plays an important anti-hypertrophic role in the heart and it has three major targets involved in the hypertrophic processes such as *Ncx1*, *Nfatc3*, and *Camk2d* (Fig. 6).

NCX1 is a bidirectional transporter controlled by membrane potentials and Na^+^ and Ca^2+^ gradients across plasma membrane [29]. At physiological condition in the heart, NCX1 is the major exchanger by which Ca^2+^ is extruded from the cell. Under pathological conditions such as cardiac hypertrophy [30] and ischemic/reperfusion injury [31], however, NCX1 predominantly function in reverse mode due mainly to the elevated [Na^+^]_i_ [32,33], mediating Ca^2+^ influx. Increased expression of the exchanger has been well documented in human and mouse models of hypertrophy and heart failure [34–36] and NCX1 transgenic mice exhibited hypertrophy and heart failure [37], suggesting the significant correlation between elevation of the NCX1 expression and the magnitude of cardiac pathology. Recently, evidence showed that both genetic ablation [31] and pharmacological inhibition [38,39] of NCX1 can have cardioprotective effects possibly due to prevention of the excessive Ca^2+^ overload by inhibiting the reverse mode. miRNAs such as *miR-1* [40,41] and *miR-214* [42], display anti-hypertrophic or cardioprotective effects by directly targeting 3′-UTR of *Ncx1*. Similar to the studies described above, in our studies *miR-185* blunted cardiomyocyte hypertrophy (Figs. 1A-1D). Inhibition of NCX1 by 1 μM SEA0400 (2-[4-[(2,5-difluorophenyl) methoxy] phenoxy]5-ethoxyaniline), a potent NCX1 inhibitor [43], also reduced cardiomyocyte size and expression of the hypertrophic markers, *ANF* and *BNP*, to a similar extent (Figure I in S1 File), demonstrating that anti-hypertrophic effect of *miR-185* is due, in part, to repression of *Ncx1*.

Calcineurin-NFAT signaling [44–46] and CaMK [44] signaling are the well-known Ca^2+^-dependent pathways. Among the five members of NFAT family, NFATc3 is an essential downstream effector of calcineurin-regulated cardiac hypertrophy [47]. CaMKII is a multifunctional protein kinase that has been implicated in cardiac hypertrophy and heart failure [48]. Among four isoforms (α, β, δ and γ) of CaMKIIs, CaMKIIδ is the predominant form in the heart [49]. Overexpression of CaMKIIδ induced pathological cardiac hypertrophy and dilated cardiomyopathy [50,51], while CaMKIIδ-null mice prevents cardiac hypertrophy and fibrosis in the aortic-constriction model [52]. CaMKII exerts its effect on cardiac hypertrophy through selective phosphorylation of class II histone deacetylase 4 (HDAC4) and the subsequent activation of MEF2, which is sufficient to promote hypertrophic gene expression [53]. In the present study, we demonstrated that *miR-185* leads to inhibition of these Ca^2+^-activated hypertrophic pathways by simultaneously targeting *Camk2d* and *Nfatc3* and the reduction of their activities.

Our cardiac-specific GSA recapitulated the previously known miRNA-pathways, including *miR-486* in PI3K-Akt signaling (*p*-value: 1.59E-03) [24], *miR-17–92* in TGF-β signaling (*p*-value: 6.34E-03) [54] and *miR-378* in MAPK signaling (*p*-value: 1.76E-02; S2 Table) [9]. With regard to *miR-185*, pathways for TGF-β, BAD and VEGF were also predicted to be closely linked to *miR-185* targets (Figure J in S1 File and S2 Table), implying that *miR-185* may also affect multiple signal transductions in the heart and may play additional roles in cardiac pathogenesis. These novel miRNA-pathway associations and multiple predicted targets shown in S2 Table will be further investigated.

The 3′-UTR of mRNA can be bound by multiple miRNAs that may in turn bring about synergistic repression of the target mRNA. In the cardiac hypertrophy signaling network (Figure C in S1 File), we also found a high degree of miRNA co-targeting to a single gene, supported by previous findings. For example, it has been reported that *Igf1r* is a target of *miR-1* [55], *miR-139* [56], *miR-378* [57], *miR-99a* [58], and *miR-497* [59]. *Ncx1* has been validated as a target of *miR-1* [40] and *miR-214* [42]. *Pten* is directly targeted by *miR-486* [24], *miR-29a* [60], and *miR-141* [61]. These lines of evidence indicate that the cardiac hypertrophy signaling pathway is under tight regulation by multiple miRNAs through redundant targeting.

*miR-185* is encoded within an intron of the *Tango2* gene (also known as *T10*) in the 22q11.2 region. DiGeorge/velocardiofacial syndrome, one of the most common human genetic deletion disorders, with a frequency of nearly one in 3,000 children, results from a microdeletion (mostly 3 Mb in size) within the same band [62,63]. It has been reported that nearly three-quarters of the patients have congenital heart disease (CHD), which is a major cause of morbidity and mortality associated with the syndrome. Interestingly, *miR-185* is the most representative down-regulated miRNA, implying that depletion of *miR-185* substantially contributes to the cardiac defects in the syndrome [64].

The previous studies have also identified additional *miR-185* target genes, such as *RhoA*, *Cdc42*, *and Stim1* [65–69], that are pro-hypertrophic in the heart, suggesting further that *miR-185* is a strong anti-hypertrophic miRNA *in vivo* and a potent therapeutic target for cardiac diseases. However, it remains to be seen whether the delivery of *miR-185* or transgenic over-expression of *miR-185* attenuates cardiac hypertrophy *in vivo*.

In conclusion, the present study shows novel evidence that *miR-185* acts as a key regulator of cardiac hypertrophy by targeting three major genes involved in Ca^2+^-associated pathological hypertrophy.

## Supporting Information

S1 File Supplementary Figures and Tables. (DOCX)

S1 Table Heart-expressed miRNAs and mRNAs. (XLSX)

S2 Table Complete list of miRNA-function associations and predicted miRNA targets in the gene sets. (XLSX)
