# Supplementary material for: miR-185 Plays an Anti-Hypertrophic Role in the Heart via Multiple Targets in the Calcium-Signaling Pathways
Source: PLoS One. 2015 Mar 13;10(3):e0122509. doi: 10.1371/journal.pone.0122509 (PMC4358957; doi:10.1371/journal.pone.0122509)
Supplement: S1 File — (DOCX) [file pone.0122509.s001.docx]

**Supporting Information**

**miR-185 plays an anti-hypertrophic role in the heart via multiple targets in the calcium-signaling pathways**

Jin Ock Kim^1^, Dong Woo Song^1^, Eun Jeong Kwon^1^, Seong-Eui Hong^1^,

Hong Ki Song^1^, Choon Kee Min^1^ and Do Han Kim^1*^^[[1]](#footnote-1)^†

^1^School of Life Sciences and Systems Biology Research Center,

Gwangju Institute of Science and Technology (GIST), Gwangju, Korea

*Corresponding author

E-mail: [dhkim@gist.ac.kr](mailto:dhkim@gist.ac.kr) (DHK)

**Materials And Methods**

**Pathway databases**

To generate a collection of gene sets, the following databases were utilized: Kyoto Encyclopedia of Genes and Genomes (KEGG) pathway database, Wiki Pathways, Molecular Signature Database (MSigDB) v2.5, Ambion GeneAssist (GA) Pathway Atlas, OMIM Morbid map and Ingenuity Pathway Analysis (IPA).

**Heart-expressed miRNAs and mRNAs**

Since miRNA-mRNA base-paring is highly dependent on the identity and quantity of miRNAs and mRNAs in particular tissues, we utilized potential miRNA-mRNA base paring of 143 consensus heart-expressed miRNAs (97 miRNA families based on the seed identity) from the literatures [1–6] and 10,451 heart-expressed mRNAs derived by RNA-Seq [7]. We determined heart-expressed mRNAs based upon their expression levels in mouse with four different conditions such as sedentary, swim, TAC and sham, since gene expression levels are different in the different cellular conditions. Expression value of each gene from RNA-Seq is normalized to reads per kilobase of exon model per million mapped reads (RPKM), as described previously [7]. Although there is currently no gold standard to determine the threshold for gene expression in the heart, we decided to use the double threshold values of ≥2 average RPKM and ≥50 average read count in either one of the four conditions from RNA-Seq data. This cutoff restricted the heart-expressed genes to 10,451, in concordance with the current estimation of ~10,000 heart-expressed genes [8].

**Cross-species miRNA target prediction**

The entire sets of miRNA-mRNA target prediction were taken from TargetScanHuman (Version 5.1, release 2009/April, http://www.targetscan.org/vert_50) and TargetScanMouse (Version 5.1, release 2009/April, http://www.targetscan.org/mmu_50). For cross-species miRNA target predictions, we presumed the potential target of miRNA, when the target gene embraces at least one conserved or non-conserved seed site for miRNA with maximum context score percentile (CSP) greater than 50 in human and mouse. Official gene symbol identical in both species was used to contrast gene by gene across two species, since it is a clean and simple way to identify orthologous pairs (80.57% was identical in our analysis based on Entrez Gene). miRNAs were grouped into families based on their seed identity and we only focused on the highly conserved miRNA families, since their functions can be conserved more across the species.

**miRNA target enrichment test**

For enrichment analysis, we used the Fisher’s exact test to compute the significance of the overlap between each miRNA targets and known sets of genes with their functional and pathway information. Significant statistical analyses for associations were performed using in-house scripts written in Python , MySQL or R (www.bioconductor.org) and *p*-value was obtained from the Fisher’s exact test using 2x2 contingency table. In the analysis, we limited testing to 177 gene sets that are functionally related with the heart and contain 10 ~ 300 genes. This step is important to avoid testing too narrowly or too broadly defined functional categories. We set the relevant number of target threshold ≥4, since if the number of predicted targets in a given pathway is not enough it is hard to estimate the specific function of miRNA. Although it could be relatively modest cutoff, we did not want to miss miRNAs of which targets are small, but critical in the signaling pathways.

**Supporting Figures and Tables**

**
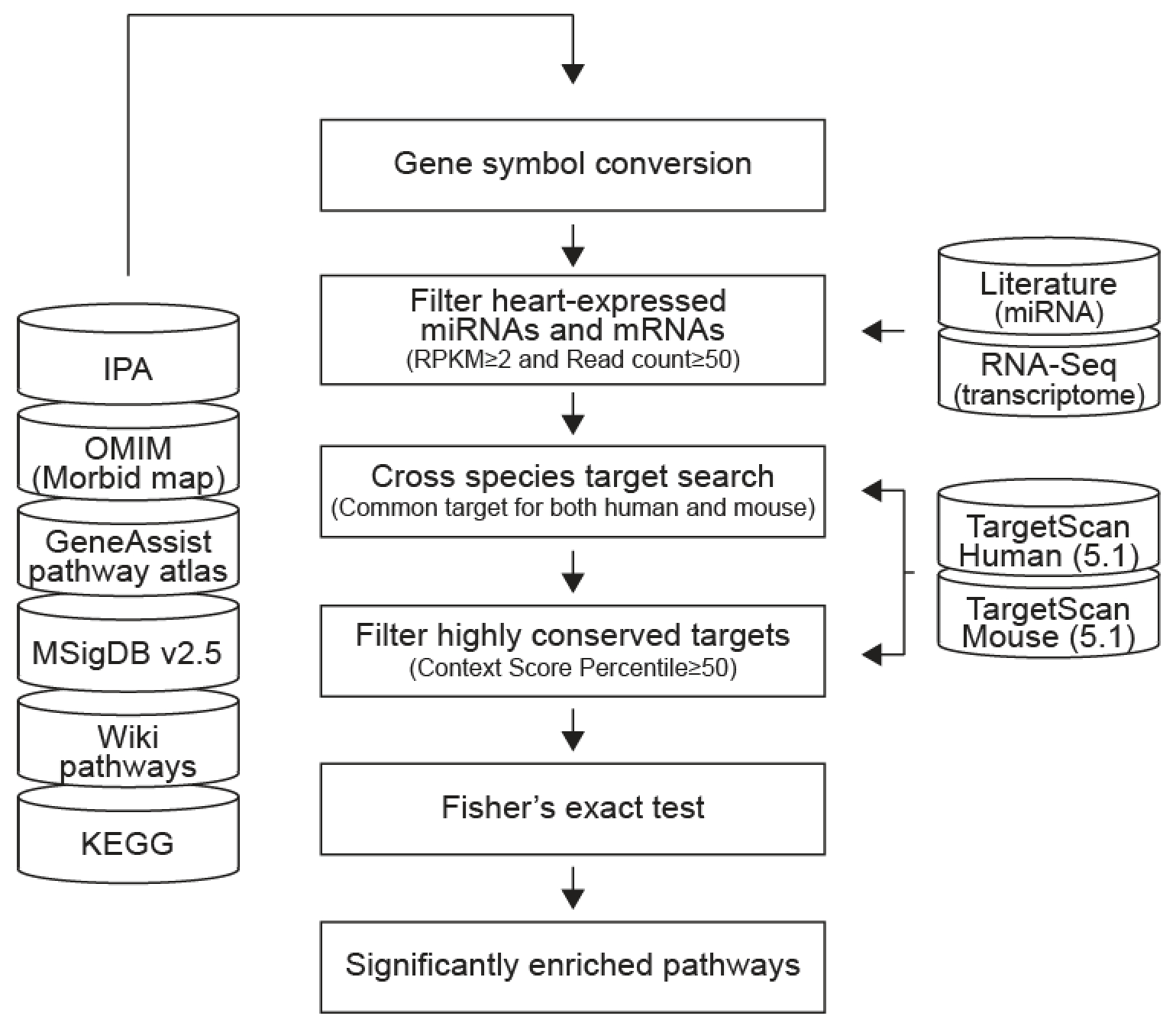
**

**Figure A. Workflow of miRNA functional enrichment analysis.** IPA, Ingenuity Pathway Analysis; OMIM, Online Mendelian Inheritance in Man; MSigDB, Molecular Signature Databases; KEGG, Kyoto Encyclopedia of Genes and Genomes; RPKM, Reads Per Kilobase per Million.


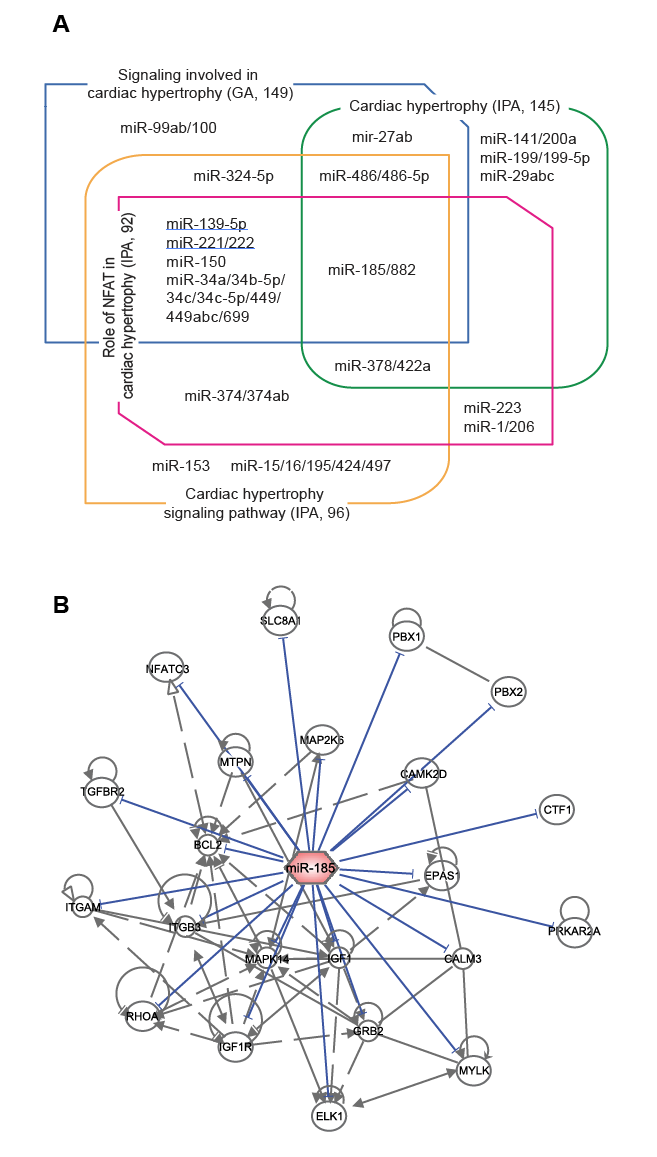


**Figure B. GSA identified 18 miRNAs significantly associated with cardiac hypertrophy signaling pathway.** (A) Eighteen miRNAs whose targets are enriched in the cardiac hypertrophy signaling pathways (database, number of genes; see Table A in S1 File for a detailed list of miRNAs and targets). GA, Ambion Gene Assist Pathway Atlas; IPA, Ingenuity Pathway Analysis. (B) The *miR-185*-target network in cardiac hypertrophy signaling pathway (Figure C in S1 File). The interaction and regulation between *miR-185* targets were screened and visualized using IPA.

**
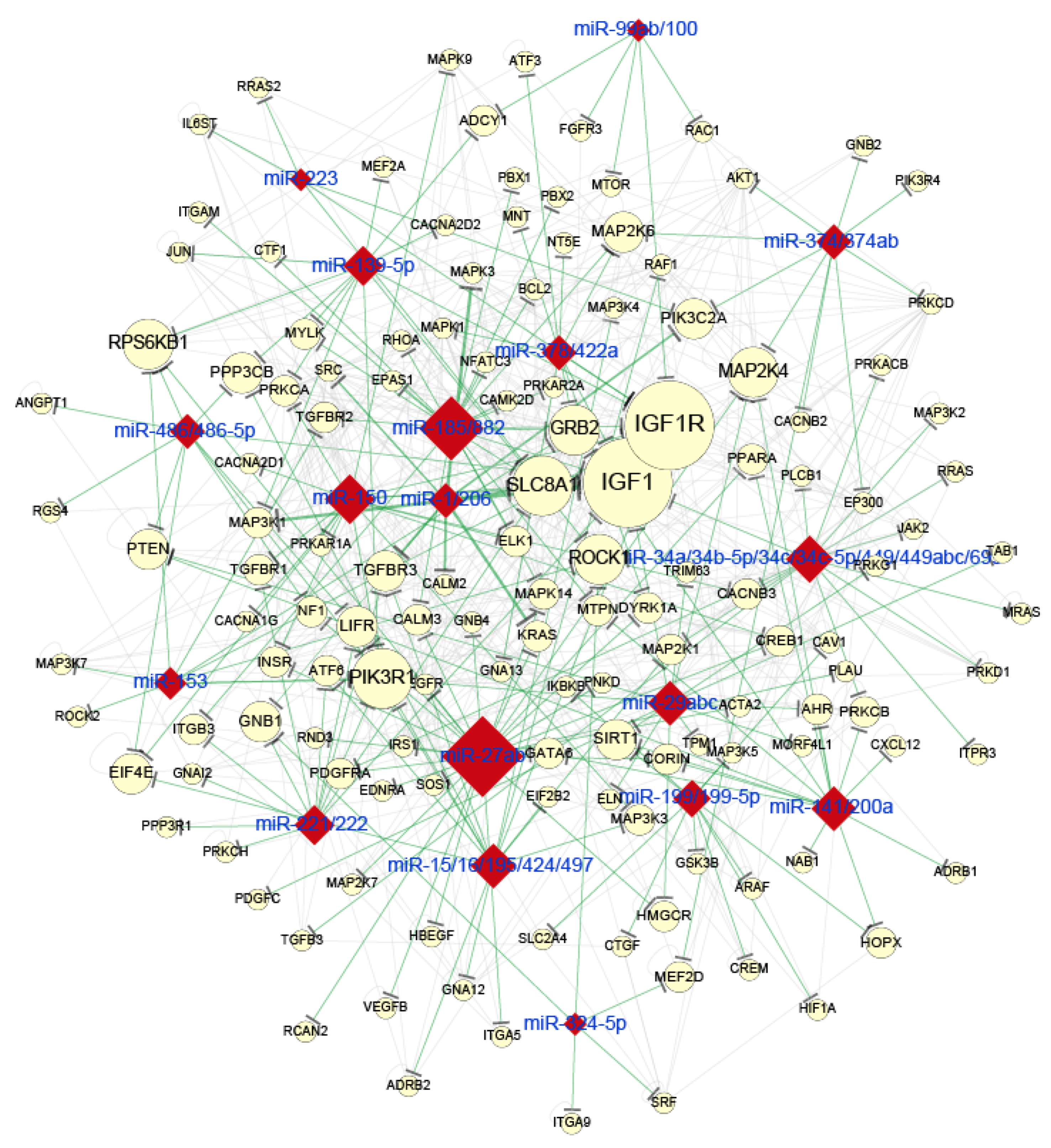
**

**Figure C. Integrated cardiac hypertrophy signaling network formed by 18 candidate miRNAs and 139 target genes.** The network was constructed through the union of 4 different cardiac hypertrophy pathways shown in Figure B in S1 File and S2 Table and visualized using Cytoscape. The edge line width is proportional to context score percentile (CSP). The size of the nodes is proportional to the degree of the relationship in the network. A larger node of miRNA indicates more connections with its targets and a larger node of target indicates more co-targeting by miRNAs. Red diamonds, miRNAs; yellow circles, target genes; green lines, predicted associations of miRNA and targets; gray lines, protein-protein interactions (PPIs) retrieved from NCBI interactome.

**
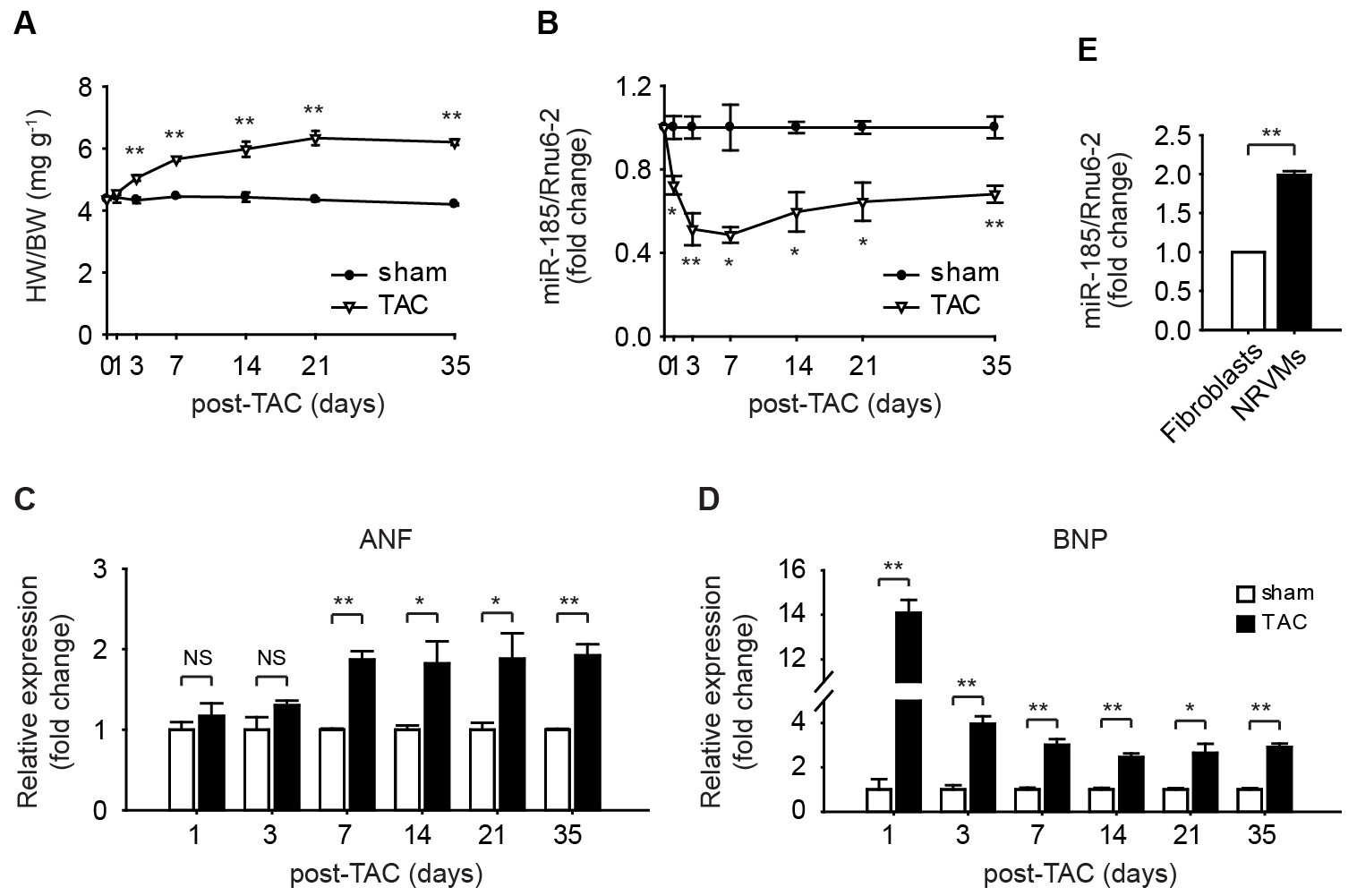
**

**Figure D. *miR-185* expression is significantly down-regulated during development of cardiac hypertrophy by TAC.** (A) Heart weight/body weight (HW/BW) ratio after sham or TAC surgery for the indicated periods. **P* < 0.05, ***P* < 0.001, vs. sham. (B) Myocardial *miR-185* expression was evaluated by qRT-PCR from mice subjected to sham or TAC surgery. (C, D) qRT-PCR data for the hypertrophic markers (*ANF* and *BNP)* measured under conditions analogous to those in (A, B). (E) Expression of *miR-185* in cardiac fibroblasts and NRVMs. miRNA expression was normalized to *U6*. All data are expressed as mean ± SEM; **P* < 0.05, ***P* < 0.001; N = 3.

**
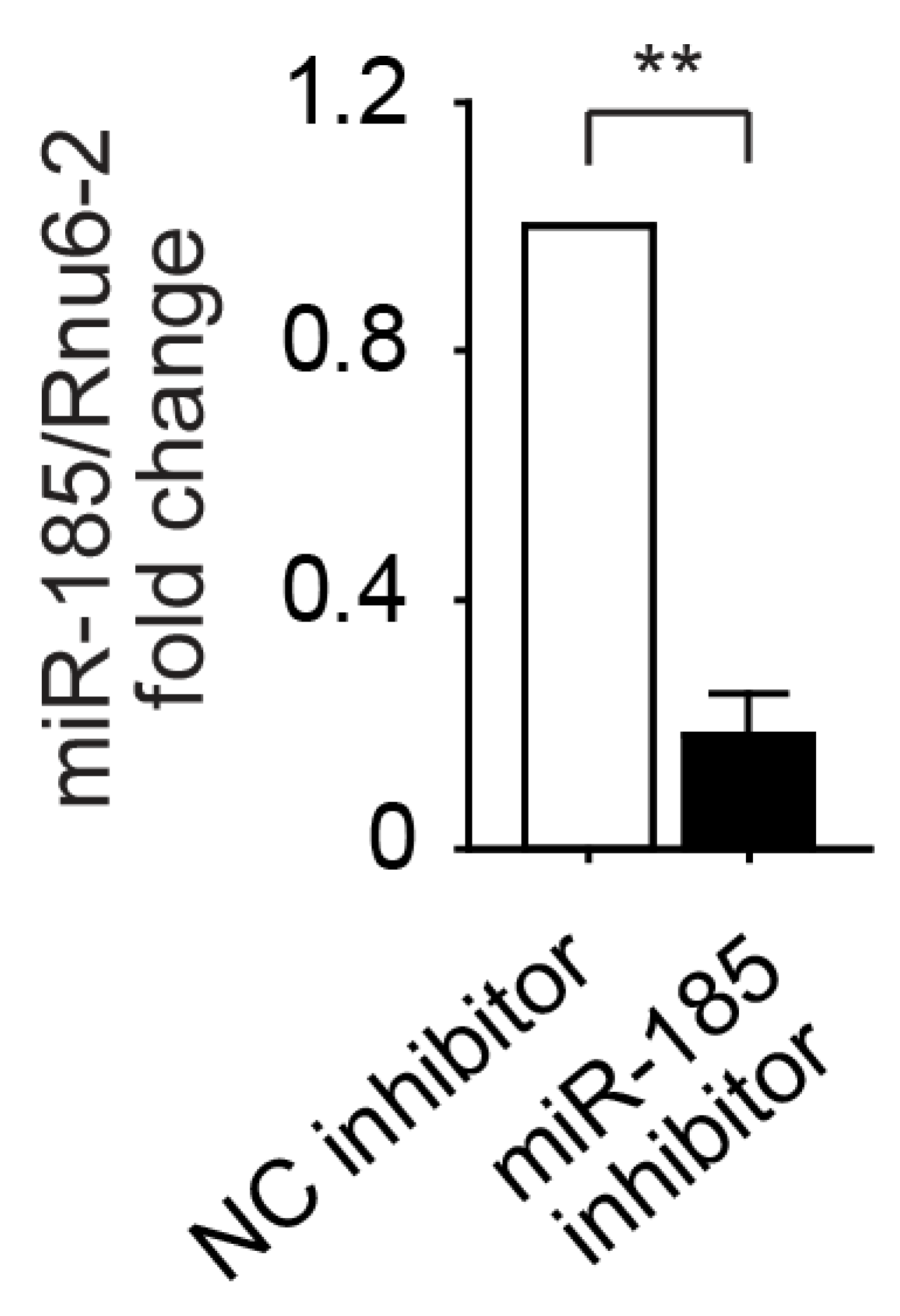
**

**Figure E. *miR-185* expression determined by qRT-PCR after transfection of *miR-185* inhibitor (100 nmol/L).** N = 3. Data represent the mean ± SEM. Statistical significance is shown as ***P*<0.001.

**
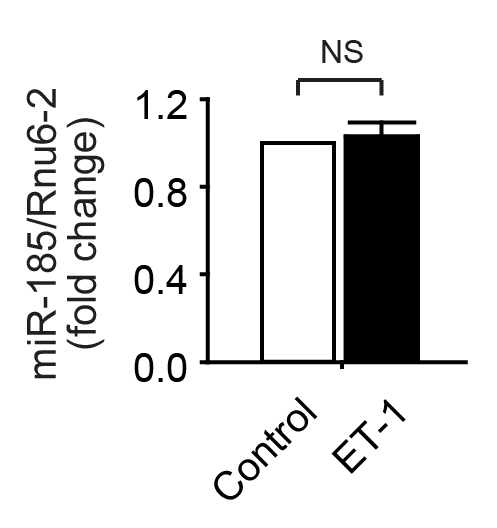
**

**Figure F. *miR-185* expression determined by qRT-PCR after treatment of ET-1 (10 nM) for 24 h.** N = 3. Data represent the mean ± SEM from three independent experiments. NS, not significant.

**
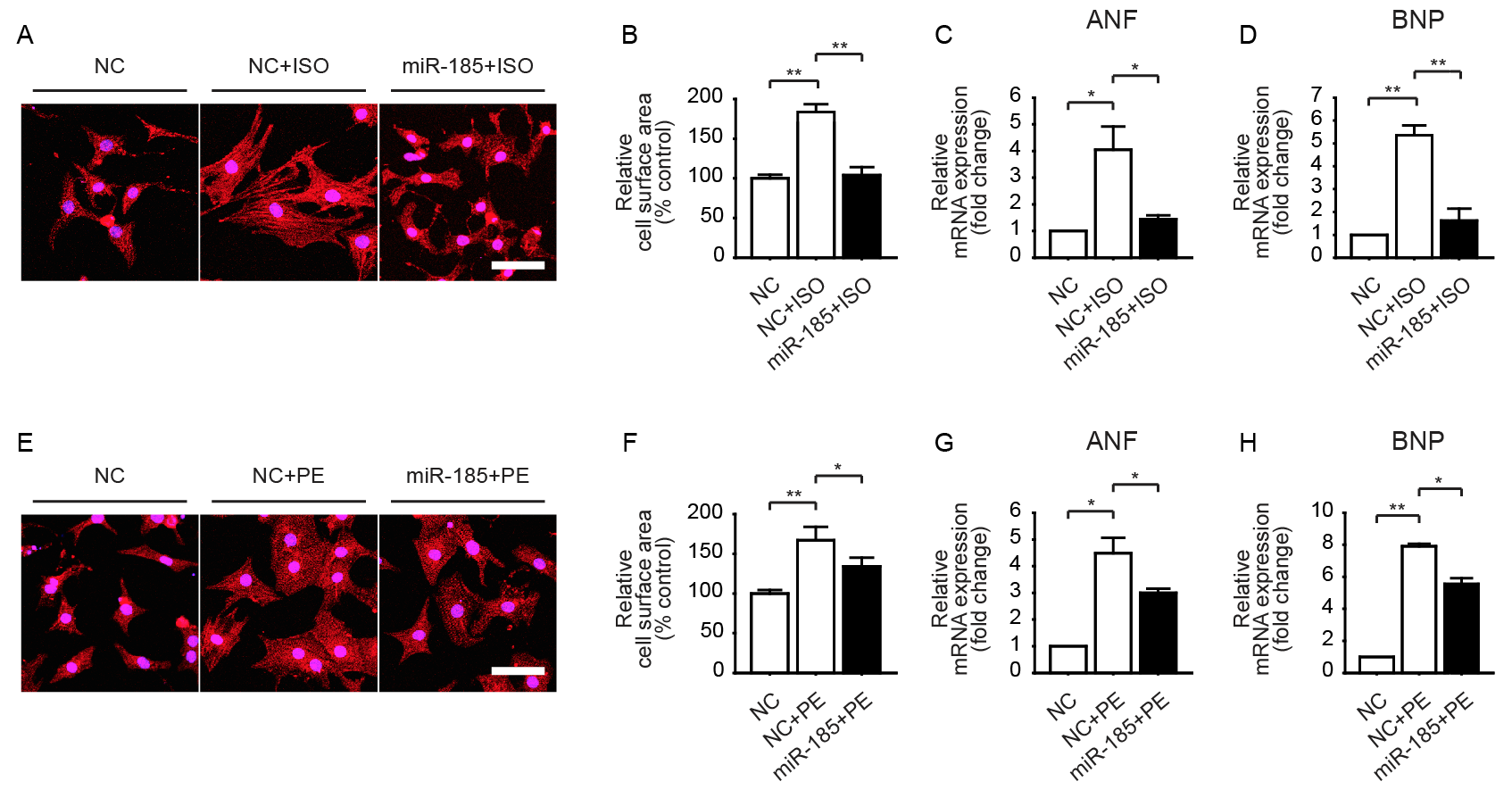
**

**Figure G. *miR-185* inhibits ISO- and PE-induced cardiomyocytes hypertrophy.** Cardiomyocytes were transfected with *miR-185* mimic or *NC* mimic. 24 h after transfection, cells were treated with ISO (10 μM) or PE (100 μM) for 48 h. (A, E) Representative images show sarcomere organization. Scale bar = 50 μm. (B, F) Cell surface area measurement. (C, D, G, H) The expression levels of ANF and BNP were analyzed by qRT-PCR. Data represent the mean ± SEM; **P* < 0.05, ***P* < 0.001; N = 3.

**
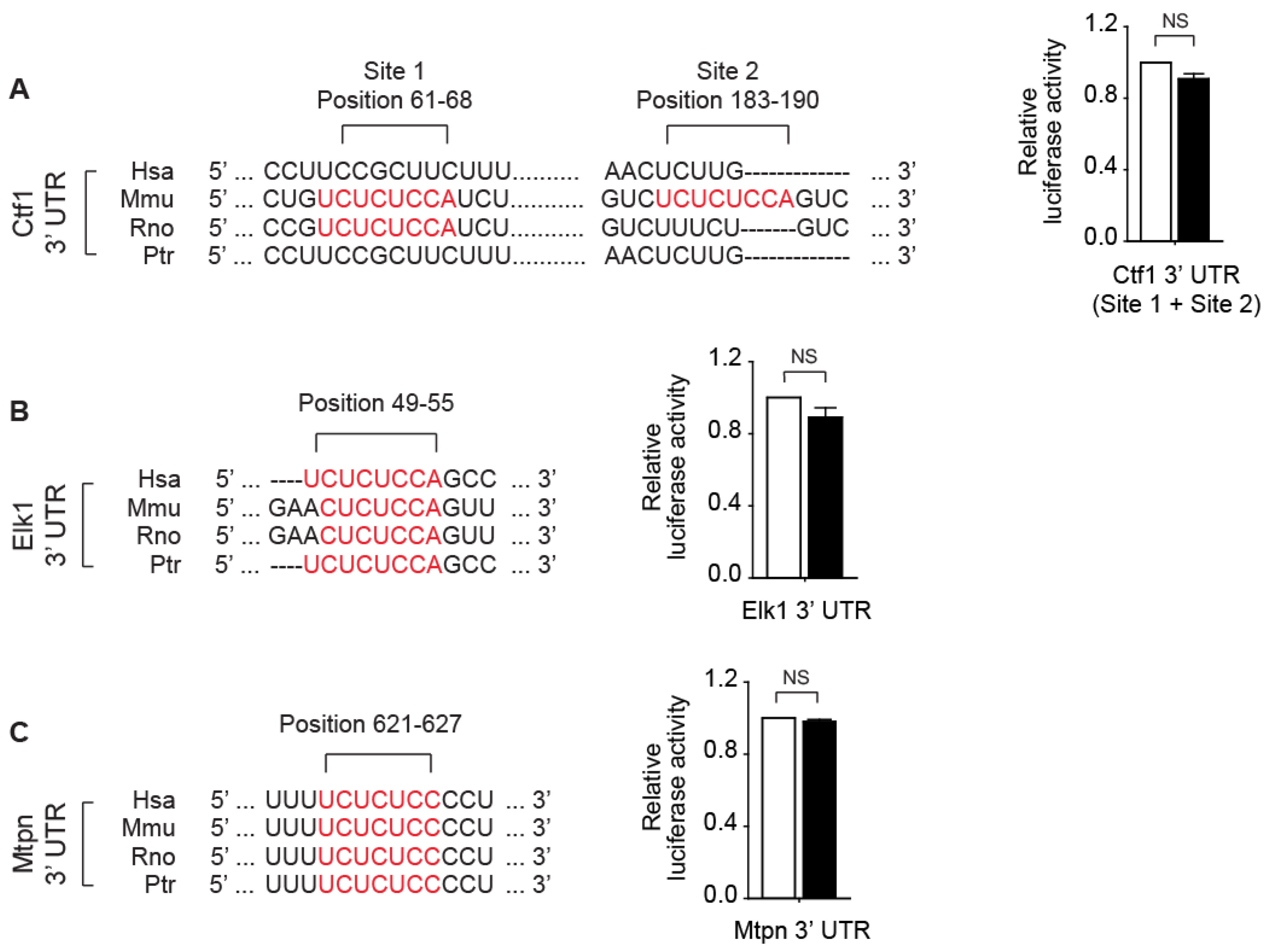
**

**Figure H. Luciferase reporter assay demonstrating that *Ctf1, Elk1,* and *Mtpn* are not the direct targets of *miR-185*.** Data represent the mean ± SEM from three independent experiments. NS, not significant; Hsa, human; Mmu, mouse; Rno, rat; Ptr, chimpanzee.


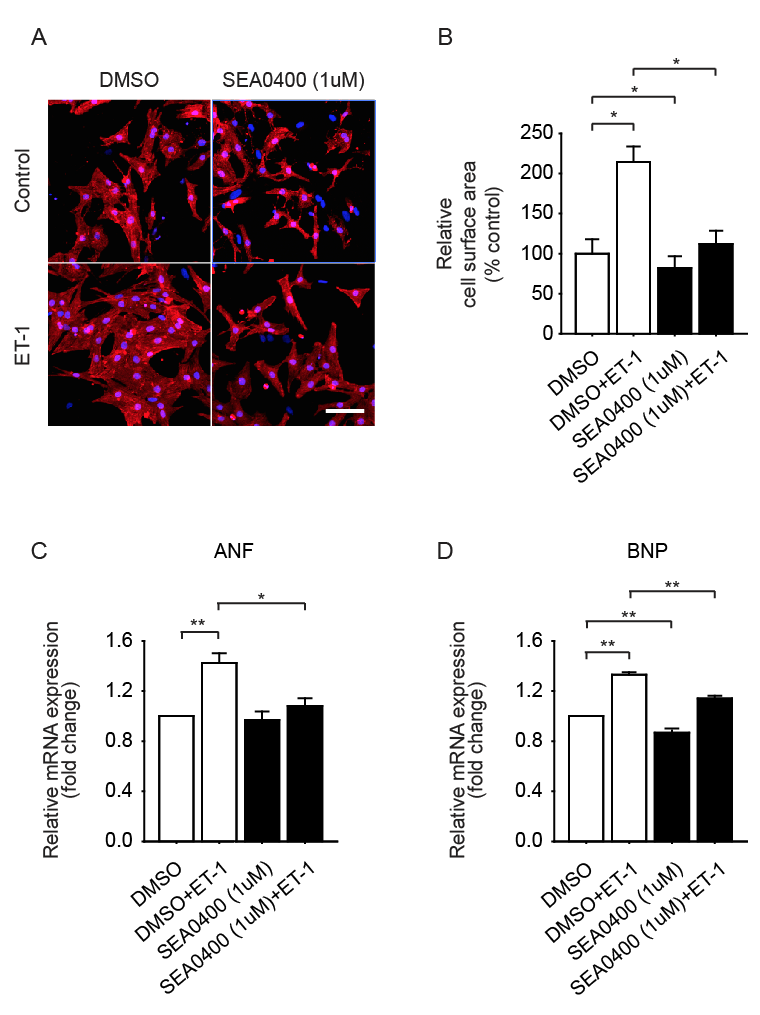


**Figure I. Pharmacological inhibition of NCX1 prevents cardiomyocyte hypertrophy.** (A) Morphological changes were observed in NRVMs stained with α-actinin antibody and Hoechst 33342 by confocal microscopy. NRVMs were stimulated with ET-1 for 24 h in the presence or in the absence of SEA0400 (1 μmol/L). Scale bar = 50 μm. Summarized data are shown in (B). (C and D) Evaluation of the influence of SEA0400 on expression of *ANF* and *BNP* at mRNA level. All data are expressed as mean ± SEM; **P* < 0.05, ***P* < 0.001; N = 3.

**
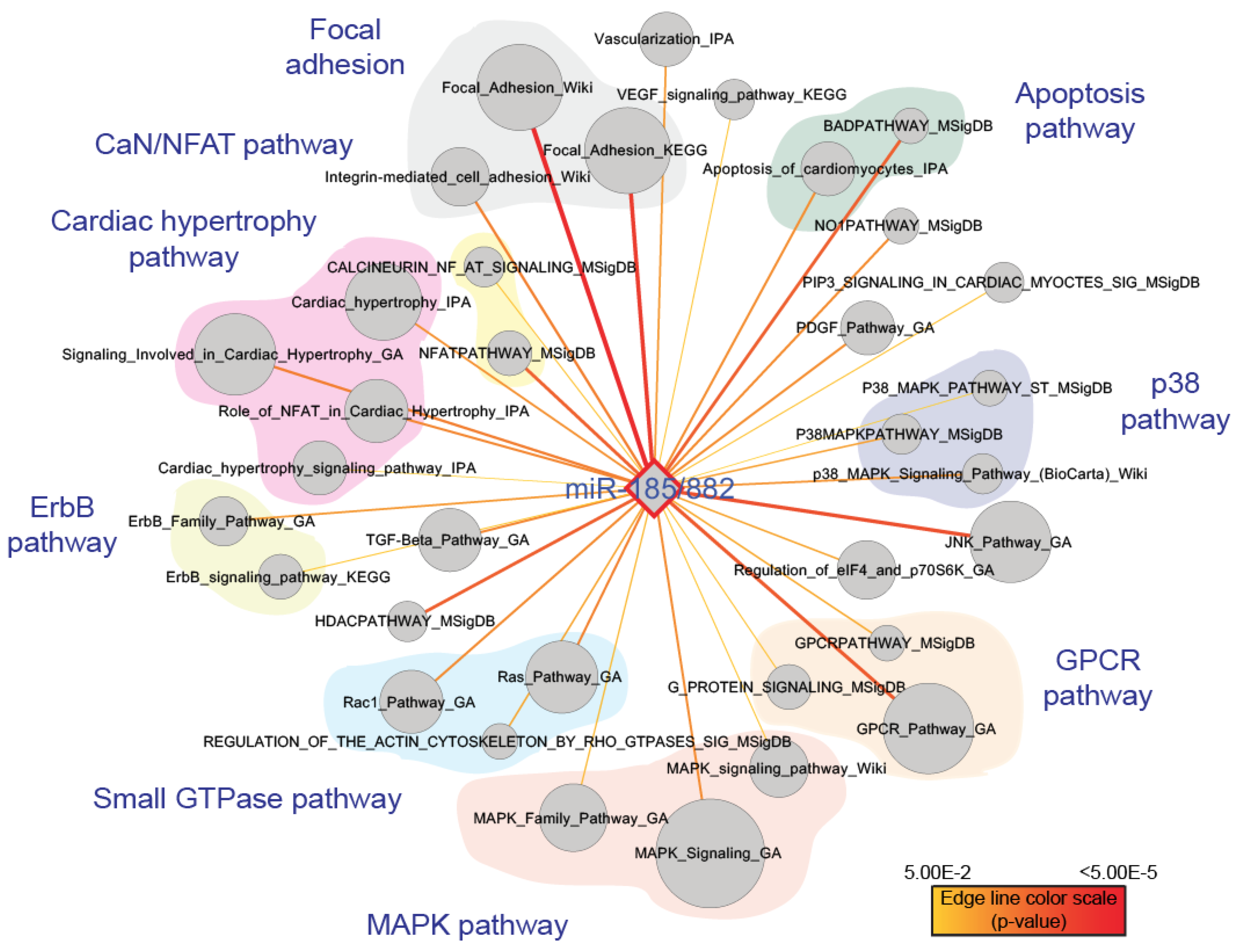
**

**Figure J. Functional spectrum of *miR-185*-regulated targets.** The size of the node is proportional to the number of targets in the gene set. The edge color represents the significance of enrichment and the deeper color represents the higher enrichment score. Functionally related pathways are marked with different color shades. GA; GeneAssist pathway atlas, IPA; Ingenuity Pathway Analysis; MSigDB, Molecular Signature Databases; KEGG, Kyoto Encyclopedia of Genes and Genomes; Wiki, Wikipathways.

**Table A. A list of miRNAs predicted to target cardiac hypertrophy signaling pathway.**

| Cardiac hypertrophy signaling pathway | miRNA family | # Targets | *p*-value |
| --- | --- | --- | --- |
| Cardiac hypertrophy signaling pathway_IPA | miR-153 | 9 | 0.00744121 |
|  | miR-324-5p | 4 | 0.0308764 |
|  | miR-34a/34b-5p/34c/34c-5p/449/449abc/699 | 10 | 0.0233174 |
|  | miR-15/16/195/424/497 | 14 | 0.03683 |
|  | miR-150 | 11 | 0.00154511 |
|  | miR-185/882 | 8 | 0.0460149 |
|  | miR-139-5p | 9 | 0.0054586 |
|  | miR-486/486-5p | 5 | 0.00910316 |
|  | miR-374/374ab | 9 | 0.0414261 |
|  | miR-221/222 | 7 | 0.0194619 |
|  | miR-378/422a | 6 | 0.011847 |
| Cardiac hypertrophy_IPA | miR-141/200a | 14 | 0.02691 |
|  | miR-199/199-5p | 11 | 0.0207382 |
|  | miR-27ab | 17 | 0.0390241 |
|  | miR-185/882 | 13 | 0.00715678 |
|  | miR-486/486-5p | 7 | 0.00316327 |
|  | miR-29abc | 14 | 0.0105488 |
|  | miR-378/422a | 7 | 0.0250291 |
| Role of NFAT in Cardiac Hypertrophy_IPA | miR-374/374ab | 9 | 0.0327537 |
|  | miR-378/422a | 6 | 0.0097135 |
|  | miR-139-5p | 8 | 0.0133193 |
|  | miR-150 | 8 | 0.0313896 |
|  | miR-223 | 5 | 0.0468615 |
|  | miR-221/222 | 8 | 0.00455914 |
|  | miR-185/882 | 10 | 0.0046874 |
|  | miR-34a/34b-5p/34c/34c-5p/449/449abc/699 | 10 | 0.017796 |
|  | miR-1/206 | 10 | 0.0197548 |
| Signaling Involved in Cardiac Hypertrophy_GA | miR-99ab/100 | 5 | 0.0000341 |
|  | miR-139-5p | 11 | 0.0131629 |
|  | miR-486/486-5p | 6 | 0.0145969 |
|  | miR-150 | 15 | 0.000908125 |
|  | miR-221/222 | 11 | 0.00340914 |
|  | miR-185/882 | 14 | 0.00350113 |
|  | miR-324-5p | 5 | 0.0370503 |
|  | miR-34a/34b-5p/34c/34c-5p/449/449abc/699 | 14 | 0.0188295 |
|  | miR-27ab | 18 | 0.0263942 |

**Table B. Primers for qRT-PCR analysis.**

| Gene | Sequence | |
| --- | --- | --- |
|  | Forward primer (5'-3') | Revers primer (5'-3') |
| ANF | ACCTGCTAGACCACCTAGAGG | GCTGTTATCTTCCGTACCGG |
| BNP | CAGCTGCCTGGCCCATCACT | ACCTCCCAGCGGCGACAGAT |
| Camk2d | CCATCACCAGAATGGGACACAG | GCATCATGGAGGCAACAGTAGAAC |
| Nfatc3 | CCTCCACAAGGCATTGAGACAC | GGACACCGATCTCACCAGCA |
| Ncx1 | GTGTTTGTCGCTCTTGGAACCTC | ACGCATCTGCATACTGGTCCTG |
| 18S | TTCTGGCCAACGGTCTAGACAAC | CCAGTGGTCTTGGTGTGCTGA |

**Supplemental References**

1. Zhang X, Azhar G, Helms S a, Wei JY. Regulation of cardiac microRNAs by serum response factor. J Biomed Sci. 2011;18: 15.

2. Ikeda S, Kong SW, Lu J, Bisping E, Zhang H, Allen PD, et al. Altered microRNA expression in human heart disease. Physiol Genomics. 2007;31: 367–373.

3. Kawahara Y, Zinshteyn B, Sethupathy P, Iizasa H, Hatzigeorgiou AG, Nishikura K. Redirection of silencing targets by adenosine-to-inosine editing of miRNAs. Science. 2007;315: 1137–1140.

4. Prasad SVN, Duan Z, Gupta MK, Surampudi VSK, Volinia S, Calin GA, et al. Unique MicroRNA Profile in End-stage Heart Failure Indicates Alterations in Specific Cardiovascular Signaling Networks. J Biol Chem. 2009;284: 27487–27499.

5. Wang K, Long B, Zhou J, Li P-F. miR-9 and NFATc3 regulate myocardin in cardiac hypertrophy. J Biol Chem. 2010;285: 11903–11912.

6. Condorelli G, Latronico MVG, Dorn GW. microRNAs in heart disease: putative novel therapeutic targets? Eur Heart J. 2010;31: 649–658.

7. Song HK, Hong S-E, Kim T, Kim DH. Deep RNA sequencing reveals novel cardiac transcriptomic signatures for physiological and pathological hypertrophy. PLoS One. 2012;7: e35552.

8. Dorn GW. MicroRNAs in cardiac disease. Transl Res. 2011;157: 226–235.

1. [↑](#footnote-ref-1)
